# In silico validation of the Autoinflammatory Disease Damage Index

**DOI:** 10.1136/annrheumdis-2018-213725

**Published:** 2018-08-04

**Authors:** Nienke M ter Haar, Amber Laetitia Justine van Delft, Kim Valerie Annink, Henk van Stel, Sulaiman M Al-Mayouf, Gayane Amaryan, Jordi Anton, Karyl S Barron, Susanne Benseler, Paul A Brogan, Luca Cantarini, Marco Cattalini, Alexis-Virgil Cochino, Fabrizio de Benedetti, Fatma Dedeoglu, Adriana Almeida de Jesus, Erkan Demirkaya, Pavla Dolezalova, Karen L Durrant, Giovanna Fabio, Romina Gallizzi, Raphaela Goldbach-Mansky, Eric Hachulla, Veronique Hentgen, Troels Herlin, Michaël Hofer, Hal M Hoffman, Antonella Insalaco, Annette F Jansson, Tilmann Kallinich, Isabelle Kone-Paut, Anna Kozlova, Jasmin Beate Kuemmerle-Deschner, Helen J Lachmann, Ronald M Laxer, Alberto Martini, Susan Nielsen, Irina Nikishina, Amanda K Ombrello, Seza Özen, Efimia Papadopoulou-Alataki, Pierre Quartier, Donato Rigante, Ricardo Russo, Anna Simon, Maria Trachana, Yosef Uziel, Angelo Ravelli, Grant Schulert, Marco Gattorno, Joost Frenkel

**Affiliations:** 1 Laboratory for Translational Immunology, University Medical Centre Utrecht, Utrecht, The Netherlands; 2 Department of Paediatric Immunology and Rheumatology, University Medical Centre Utrecht, Utrecht, The Netherlands; 3 Department of Paediatrics, Universitair Medisch Centrum Utrecht–Locatie Wilhelmina Kinderziekenhuis, Utrecht, The Netherlands; 4 Department of Pediatrics, King Faisal Specialist Hospital and Research Center, Riyadh, Saudi Arabia; 5 National Paediatric Centre for Familial Mediterranean Fever and Gastroenterology Service, Arabkir Medical Centre–Institute of Child and Adolescent Health, Yerevan, Armenia; 6 Paediatric Rheumatology Unit, Hospital Sant Joan de Déu, Barcelona, Spain; 7 Division of Intramural Research and National Institute of Allergy and Infectious Diseases, National Institutes of Health, Bethesda, Maryland, USA; 8 Departments of Paediatrics and Rheumatology, Alberta Children’s Hospital, Calgary, Alberta, Canada; 9 Department of Infection, Inflammation and Rheumatology, University College London Institute of Child Health, London, UK; 10 Rheumatology Unit, Department of Medical Sciences, Surgery and Neurosciences, University of Siena, Siena, Italy; 11 Paediatric Clinic, University of Brescia and Spedali Civili di Brescia, Brescia, Italy; 12 Department of Paediatrics, National Institute for Mother and Child Health Alessandrescu-Rusescu, Bucharest, Romania; 13 Division of Rheumatology, Ospedale Pediatrico Bambino Gesù, Rome, Italy; 14 Rheumatology Program, Division of Immunology, Boston Children’s Hospital, Harvard Medical School, Boston, Massachusetts, USA; 15 Translational Autoinflammatory Disease Section, NIAMS/NIH, Bethesda, Maryland, USA; 16 Western University Children’s Hospital, London Health Sciences Centre, London, UK; 17 Department of Paediatrics and Adolescent Medicine, Charles University, General University Hospital, Praha, Czech Republic; 18 Autoinflammatory Alliance, San Francisco, California, USA; 19 Department of Internal Medicine, Fondazione IRCCS Ca’ Granda Ospedale Maggiore Policlinico, Milano, Italy; 20 Department of Paediatric Rheumatology, AOUG Martino, Messina, Italy; 21 Département de Médecine Interne et Immunologie Clinique, Université de Lille, Lille, France; 22 Reference Centre for Autoinflammatory Diseases (CEREMAI), Versailles Hospital, Le Chesnay, France; 23 Departmentof Paediatrics, Aarhus University Hospital, Aarhus, Denmark; 24 Department of Paediatric Rheumatology, University of Lausanne, Lausanne, Switzerland; 25 Departmentof Paediatric Rheumatology, University Hospital of Geneva, Geneva, Switzerland; 26 Department of Paediatrics, University of California, San Diego, California, USA; 27 Dipartimento di Medicina Pediatrica, IRCCS Ospedale Pediatrico Bambino Gesù, Roma, Italy; 28 Paediatric Pneumology and Immunology and Interdisciplinary Centre for Social Paediatrics, Charite University Medicine Berlin, Berlin, Germany; 29 Department of Rheumatology and Immunology, Dr von Hauner Children’s Hospital, Ludwig-Maximilians-University, Munich, Germany; 30 Department of Paediatric Rheumatology and CEREMAI, Bicêtre Hospital, APHP, University of Paris Sud, Paris, France; 31 Department of Immunology, Federal Research and Clinical Centre for Paediatric Haematology, Oncology and Immunology, Moscow, Russian Federation; 32 Division of Pediatric Rheumatology, Department of Pediatrics, University Hospital Tübingen, Tübingen, Germany; 33 Division of Medicine, University College London, London, UK; 34 Department of Paediatrics and Medicine, University of Toronto and the Hospital for Sick Children, Toronto, Ontario, Canada; 35 Direzione Scientifica, Istituto Giannina Gaslini, Genova, Liguria, Italy; 36 Paediatric Rheumatology Unit 4272, Rigshospitalet, Copenhagen, Denmark; 37 Department of Paediatric Rheumatic Diseases, VA Nasonova Research Institute of Rheumatology, Moscow, Russian Federation; 38 Inflammatory Disease Section, National Human Genome Research Institute, Bethesda, Maryland, USA; 39 Pediatric Rheumatology, Hacettepe University, Ankara, Turkey; 40 Fourth Department of Pediatrics, Aristotle University of Thessaloniki, Thessaloniki, Greece; 41 Department of Paediatric Immunology–Hematology and Rheumatology Unit and IMAGINE Institute, Institution Necker-Enfants Malades Hospital and Paris Descartes University, Paris, Île-de-France, France; 42 Institute of Paediatrics, Fondazione Policlinico Universitario A Gemelli, Università Cattolica Sacro Cuore, Rome, Italy; 43 Servicio de Inmunología/Reumatología, Hospital de Pediatria Juan P Garrahan, Buenos Aires, Argentina; 44 Department of General Internal Medicine, Radboud Expertise Centre for Immunodeficiency and Autoinflammation, Radboud University Medical Centre, Nijmegen, The Netherlands; 45 Paediatric Immunology and Rheumatology Referral Centre, First Paediatric Clinic, Aristotle University of Thessaloniki, Thessaloniki, Greece; 46 Department of Paediatrics, Meir Medical Centre, Kfar Saba, Israel; 47 Institution Università degli Studi di Genova and G Gaslini Institute, Genova, Italy; 48 Division of Rheumatology, Cincinnati Children’s Hospital Medical Center, Cincinnati, Ohio, USA; 49 Department of Pediatrics, University of Cincinnati, Cincinnati, Ohio, USA

## Abstract

**Introduction:**

Autoinflammatory diseases can cause irreversible tissue damage due to systemic inflammation. Recently, the Autoinflammatory Disease Damage Index (ADDI) was developed. The ADDI is the first instrument to quantify damage in familial Mediterranean fever, cryopyrin-associated periodic syndromes, mevalonate kinase deficiency and tumour necrosis factor receptor-associated periodic syndrome. The aim of this study was to validate this tool for its intended use in a clinical/research setting.

**Methods:**

The ADDI was scored on paper clinical cases by at least three physicians per case, independently of each other. Face and content validity were assessed by requesting comments on the ADDI. Reliability was tested by calculating the intraclass correlation coefficient (ICC) using an ‘observer-nested-within-subject’ design. Construct validity was determined by correlating the ADDI score to the Physician Global Assessment (PGA) of damage and disease activity. Redundancy of individual items was determined with Cronbach’s alpha.

**Results:**

The ADDI was validated on a total of 110 paper clinical cases by 37 experts in autoinflammatory diseases. This yielded an ICC of 0.84 (95% CI 0.78 to 0.89). The ADDI score correlated strongly with PGA-damage (r=0.92, 95% CI 0.88 to 0.95) and was not strongly influenced by disease activity (r=0.395, 95% CI 0.21 to 0.55). After comments from disease experts, some item definitions were refined. The interitem correlation in all different categories was lower than 0.7, indicating that there was no redundancy between individual damage items.

**Conclusion:**

The ADDI is a reliable and valid instrument to quantify damage in individual patients and can be used to compare disease outcomes in clinical studies.

## INTRODUCTION

Autoinflammatory diseases (AID) are characterised by seemingly unprovoked, recurrent episodes of inflammation caused by activation of the innate immune system. The four most common monogenic AIDs are cryopyrin-associated periodic syndromes (CAPS), tumour necrosis factor receptor-associated periodic syndrome (TRAPS), mevalonate kinase deficiency (MKD) and familial Mediterranean fever (FMF).^1,2^ Chronic inflammation in AIDs may cause irreversible damage in multiple organ systems, such as visual loss, deafness, joint restriction and amyloidosis.^3^

Even though targeted therapy for these AIDs has become available,^4–6^ permanent damage may still accumulate before diagnosis or start of therapy. Furthermore, the majority of studies on new biological therapies for AIDs are recently initiated, with limited follow-up, hence the potency of these drugs to prevent or stop the development of damage is not yet known.^3,7,8^ The Autoinflammatory Disease Damage Index (ADDI) has been developed to enable assessment of the long-term burden of AIDs in a standardised manner, as a comprehensive tool measuring damage in patients with AIDs.^5^ Although developed for the four main monogenic AIDs, the ADDI may potentially also be useful in other diseases with autoinflammatory features.^9,10^

To properly validate a damage index such as the ADDI, several aspects are important: reliability, content validity, face validity, criterion validity and construct validity.^11^ A reliable index means that for a given patient, different observers will give the same score; this can be assessed by calculating the interobserver variability (intraclass correlation coefficient, ICC). Content validity tests whether the content of the index truthfully reflects the subject the index applies to. Face validity is the subjective impression whether a test measures the intended phenomenon. Criterion validity tests whether an index is as good as the gold standard. Construct validity consists of convergent and discriminant validity: convergent validity determines whether an index correlates to a similar index (eg, whether the ADDI correlates to other indices of damage or impairments in daily living), whereas discriminant validity determines whether the index is different from a dissimilar index (eg, the ADDI should not correlate with indices of disease activity).

Continuously during development and validation of the ADDI, content validity, face validity and adherence to the OMERACT principles (truth, discrimination and feasibility) were assessed.^12–14^ As a reference standard for disease damage in AIDs is lacking, criterion validity cannot be determined. Physician Global Assessment (PGA) of damage can be considered the best alternative for a gold standard, but it is not a validated measure. Therefore, we decided to use the PGA-damage to assess construct validity rather than criterion validity. Hence, in this study we aimed to investigate the reliability and construct validity, using paper clinical cases of patients with FMF, CAPS, TRAPS and MKD, designed to ensure that all the damage items were adequately covered.

## METHODS

### Development of the validation plan

Together with an experienced methodologist (HvS), a validation plan was developed. Paper clinical cases were based on real patient data, but modified to protect patient privacy and to ensure that all damage items would be sufficiently represented and different degrees of damage could be tested. Using a pilot with a limited number of cases and expert participants, a preliminary ICC was determined and the final number of cases required for the validation was calculated. All expert physicians who participated in the development of the ADDI (top 40 enrollers in the Eurofever Registry and nine experts from the Americas) were invited to participate in the validation process. One expert involved in the development of paper cases (JF) did not take part in the scoring.

### Development of the cases

The cases for validation of the ADDI were derived from anonymised clinical data of patients with confirmed FMF, CAPS, TRAPS and MKD included in the European-based online Eurofever Registry.^15,16^ All physicians involved in the Eurofever project (Executive Agency for Health and Consumers, Project No 2007332) were asked to complete follow-up data on patients they had entered in the registry. The registry collects detailed information on all potential organ involvement as well as general features of AIDs. To cover a wide case mix, expert physicians from the Americas were asked to submit their anonymous patient data using a preformed template. The patient information retrieved from the Eurofever Registry and American cases served as a resource for paper clinical case scenarios. Cases were modified to ensure that each ADDI item was represented at least four times. Precautions were made to provide a similar number of cases for each disease and to have cases with different grades of disease activity and damage. All paper cases were checked for comprehensiveness and realistic character by one expert (JF).

### Case distribution

The case summaries were distributed via a web-based survey, in which experts completed the ADDI, estimated the degree of disease damage and disease activity using a 10-point PGA-damage and PGA-activity, respectively, and could provide comments. The distribution of cases followed the ‘observer-nested-within-subject’ design, meaning that a large group of experts all scored a subset of the cases.^11^ Each group of four experts scored 10 cases each, a minimum of three doctors was needed per group to calculate the ICC. Additional experts were asked to complete the survey when necessary. An equal proportion of adult and paediatric physicians was ensured in each participant group. Furthermore, each group contained four doctors from different countries and centres.

### Definition of damage

Damage is defined as persistent or irreversible change in structure or function, which is present for at least 6 months. Damage items should not be scored if they are attributed to ongoing disease activity. Damage may be the result of prior disease activity, complications of therapy or comorbid conditions that developed after the onset of AID signs and symptoms. If damage has been present for longer than 6 months, but later resolves, it should still be scored in order to capture the damage that was present in the individual for that time period. This definition can be found within in the ADDI in earlier versions of the damage index.^12^

### Statistical analysis

Statistical analyses were performed in IBM SPSS Statistics V.21. The total score of the ADDI is the sum of points given for all categories. The ICC was determined to assess the reliability of the damage index as a whole, as well as for the eight categories and all individual items. The ICC determined absolute agreement, for example, whether two different physicians give the exact same score for a given patient, and considered single measures, indicating reliability of a single observer.^11^ The ICC was also assessed for the PGA-damage and the PGA-activity, in order to determine whether these measurements would be sufficiently reliable to test construct validity. An ICC of 0.8 or higher was considered indicative for excellent reliability.^11,17^ Cronbach’s alpha was used to determine possible redundancy of different items (eg, whether two items would score the same damage). An interitem correlation of more than 0.7 was considered to indicate redundancy.^18^ A Spearman rank test was used to assess discriminant and convergent validity, correlating the ADDI to PGA-activity and PGA-damage, respectively. A Spearman rank test with r=0.1–0.3 was considered weak, r=0.3–0.5 was considered moderate and r>0.5 was considered strong.^19^

### Discussion on the items and definitions

A small team (NMtH, ALJvD, JF) discussed all items with an ICC below 0.7. This discussion encompassed possible explanations for a low score (eg, unclear definition of an item or the lack of a growth chart hampering easy scoring of growth failure). Further, based on experts’ comments and suggestions during the scoring, possibilities to improve the item and/or definition were discussed. The initial and refined items were proposed to all experts via a web-based survey and subsequently discussed in an open face-to-face meeting at the Paediatric Rheumatology Congress in Athens (PReS 2017). Consensus was considered achieved if more than 70% of experts agreed.

## RESULTS

### Pilot

A pilot study with 15 paper cases was completed by four experts. This yielded a preliminary ICC of 0.85 (95% CI 0.70 to 0.94), which implied that a minimum of 90 cases would be needed for the validation of the ADDI. We therefore decided to assign 110 cases to the experts.

### Collection of cases

A total of 120 patients whose follow-up had been documented in the Eurofever Registry were identified, and an additional 20 cases were submitted by non-European experts. By selecting and combining case information, a total of 110 cases were compiled from these 140 cases. The final paper clinical cases included 29 patients with CAPS, 27 with TRAPS, 29 with FMF and 25 with MKD.

### Validation

In total, 37 of 44 participants responded. In 10 groups at least three participants responded, which led to 100 cases that could be used for the analyses. Due to insufficient response in one group, these 10 cases could not be used. Each item received a non-zero score (indicating presence of that item) at least 18 times.

### Intracluster correlation coefficient

The ICC of the ADDI was 0.84 (95% CI 0.78 to 0.89). This indicates good inter-rater reliability. The ICCs per disease, for different organ systems and the individual damage items are shown in table 1. The highest ICC was found for the item ‘hearing loss’ (0.86, 95% CI 0.81 to 0.90) exceeding the overall ICC, the lowest ICC was found for the item ‘puberty delay’ (0.29, 95% CI 0.16 to 0.43).

### Construct validity

**Figure 1:**
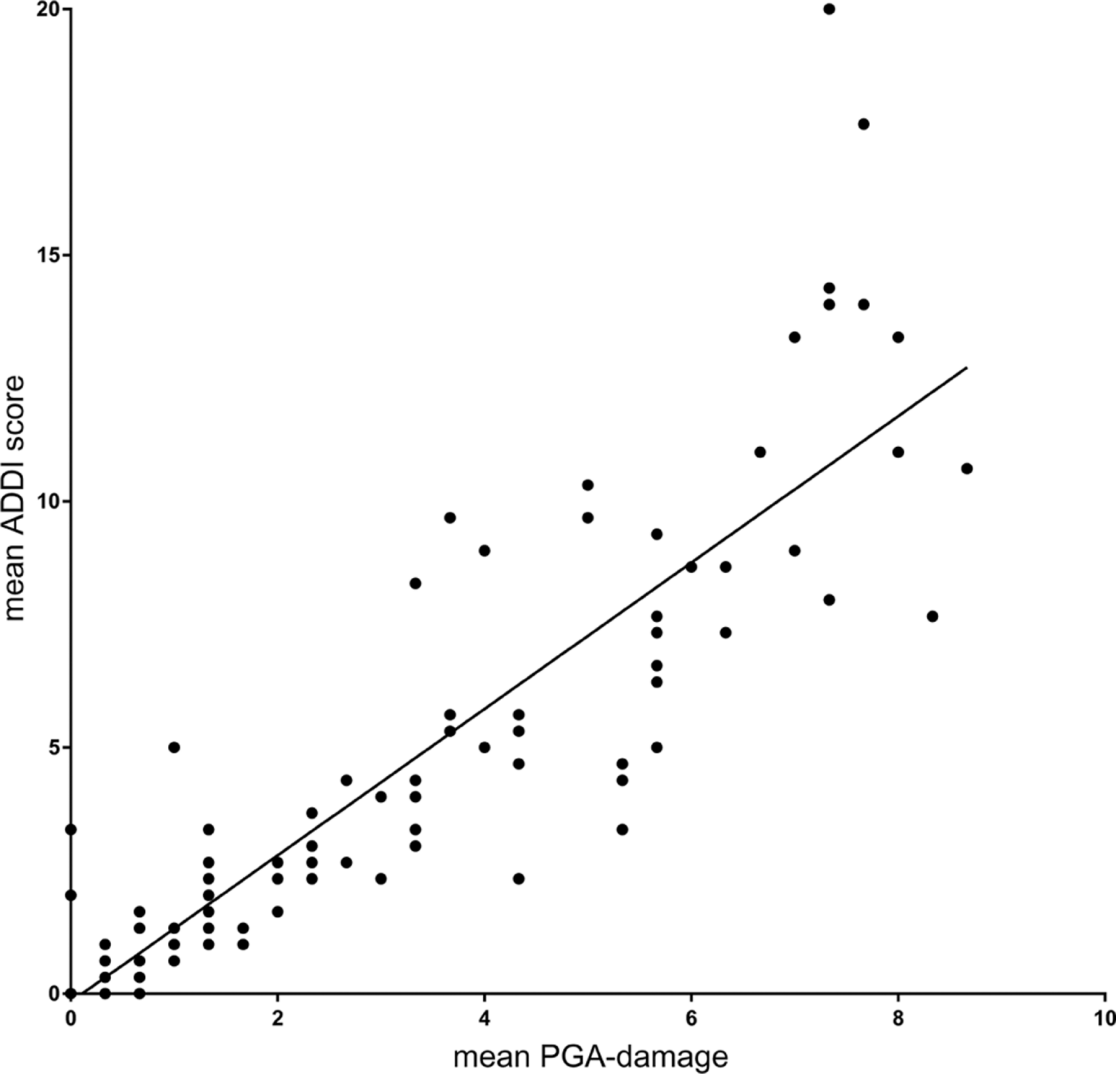
Correlation of the mean ADDI score and the mean score of damage (PGA-damage) per case, assessed by at least three observers. Each dot represents a patient case. The line indicates the correlation. ADDI, Autoinflammatory Disease Damage Index; PGA, Physician Global Assessment.

**Figure 2:**
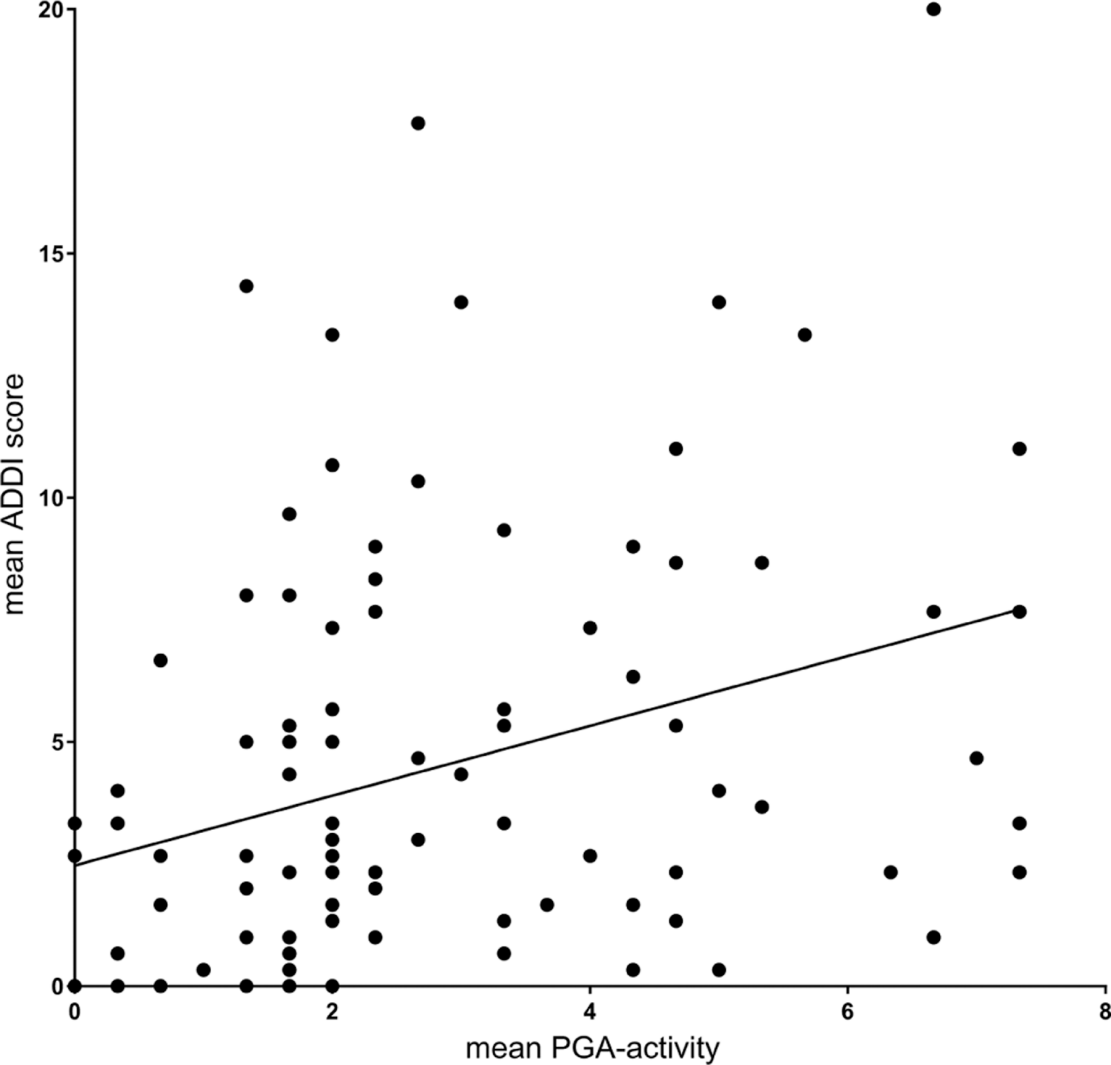
Correlation of the mean ADDI score and the mean score of activity (PGA-activity) per case, assessed by at least three observers. Each dot represents a patient case. The line indicates the correlation. ADDI, Autoinflammatory Disease Damage Index; PGA, Physician Global Assessment.

The ICCs of PGA-damage (0.75, 95% CI 0.67 to 0.81) and PGA-activity (0.62, 95% CI 0.52 to 0.71) were considered sufficiently reliable to determine construct validity. A strong relation was found between the score of the ADDI and PGA-damage (Spearman’s r=0.92, 95% CI 0.88 to 0.95, p<0.001, see figure 1). This correlation coefficient indicates that an increase in the ADDI score is strongly associated with an increase in the total estimated damage. The relation between disease activity (PGA-activity) and the ADDI score was much weaker (Spearman’s r=0.40, 95% CI 0.21 to 0.55, p<0.001, see figure 2), indicating that the ADDI is not primarily driven by disease activity.

### Interitem correlation

In order to assess whether items had too much overlap, interitem correlation was determined using Cronbach’s alpha. Of specific interest was the interitem correlation between cognitive impairment (mainly relating to adult patients or adolescents) and developmental delay (mainly relating to paediatric patients), as the experts worried that these might have too much overlap. The interitem correlation between cognitive impairment and developmental delay was 0.66, indicating that there was minimal redundancy. All interitem correlation matrixes can be found in online supplementary table 1a–e.

### Comments from the experts

The ADDI was considered a simple and easily applicable tool. The most important feedback during the survey included comments and uncertainties about scoring, for example, due to limited information in the case description (eg, the lack of growth charts to completely assess growth failure), unclear definitions in the ADDI (eg, whether psychiatric comorbidities are part of the item central nervous system (CNS) involvement), or doubts about the severity of organ involvement (eg, severity of visual loss). A full overview of these comments can be found in online supplementary table 2.

Other important comments comprised item scoring (suggesting a higher/lower weighting), or suggestions to refine item definitions. These suggestions were presented to all participants using an online survey. The results of this survey were subsequently discussed in a face-to-face meeting. Following this meeting, the maximum total score of the category ‘reproductive’ was limited to 2, in order to reduce sex differences in scoring of this category. Furthermore, slight changes were made in the definitions for growth failure, CNS involvement, joint restriction, puberty delay and serosal scarring (online supplementary table 2). The revised ADDI can be found in table 2.

All items were considered truthful, discriminative and feasible; however, doubts were raised about the reliability and feasibility of the scoring of musculoskeletal pain as there is no objective test to assess this. Despite that, it was considered that this particular item was sufficiently valid and very important to patients; therefore it was kept as part of the ADDI.

## DISCUSSION

This validation study demonstrates that the ADDI is a reliable tool to measure damage in the four main monogenic AIDs. Most items were considered clearly defined and easy to score. Further, the ADDI correlated well with the estimated damage and was not strongly influenced by disease activity, indicating good convergent and discriminant validity, respectively. No significant overlap was found between items, therefore all items were included in the final version of the ADDI. Some items were slightly refined, based on comments provided by the clinical experts.

This is the first validation of a disease damage index for AIDs. An ICC of 0.84 is comparable to other damage indices for rheumatic diseases, such as the Juvenile Arthritis Damage Index (ICC 0.85–0.97),^20^ Localized Scleroderma Skin Damage Index (ICC 0.99),^21^ Cutaneous Lupus Erythematosus Disease Area and Severity Index (ICC 0.86),^22^ Vasculitis Damage Index (ICC 0.94)^23^ and Combined Damage Assessment (ICC 0.78).^23^

A key strength of this validation study is the participation of adult and paediatric experts worldwide who all provided patient cases and scored the ADDI. This makes it plausible that the ADDI can be used in clinical settings involving paediatric as well as adult patients with FMF, CAPS, TRAPS or MKD. However, the fact that the AID experts scoring the cases were also involved in the development of the ADDI and the collection of patient information might have resulted in a relatively high ICC. Physicians with less knowledge of the tool or AIDs in general might encounter more difficulties interpreting the damage items and scoring the ADDI.

Another strength of this study is the development of cases, which were based on actual patient data while modifications were made to ensure a sufficient representation of all damage items. The total of 110 cases is a large number for validation, given the rarity of these diseases. However, the lack of validation in a real clinical setting is also a drawback of this study. The modification of cases could have resulted in less realistic scenarios. Additionally, scoring paper cases may be easier than applying ADDI in the clinical setting as all the information is summarised and presented in a uniform way. On the other hand, due to the nature of cases (paper clinical instead of real patients) participants may have interpreted data more ambiguously than they would in real life. Scoring anonymous cases, without knowing the patients or being able to ask additional questions, is probably harder than in daily practice. Indeed, comments of the participants reflected some of these difficulties they experienced when assessing the paper cases.

Some important issues could not yet be addressed due to the design of this validation study. The responsiveness to change, that is, whether accrued damage over time is also reflected in an increasing score of the ADDI in an individual patient, could not be determined. A long-term observational study would be needed to measure responsiveness to change and subsequently assess the minimal clinically important difference of the ADDI. Further, convergent validity of the ADDI should preferentially include correlations with scores on quality of life and functional ability, especially because the damage items in the ADDI had been selected for their impact on patients’ lives. As the information about quality of life and functional ability was lacking in the Eurofever Registry, this part of the construct validity was impossible to assess. Moreover, ideally the discriminative validity of the ADDI should be assessed by its correlation to a validated activity index, such as the Auto-Inflammatory Disease Activity Index (AIDAI).^24,25^ As we could not derive the AIDAI values from the patient data, we used PGA-activity as a surrogate marker. However, the ICC of PGA-activity was low with a broad CI, meaning that this estimate for activity as provided by the experts was not a very reliable measure. This may be explained by the characteristics of these AIDs, for example, episodes of febrile attacks with symptom-free periods in-between. Altogether, a long-term prospective study assessing the ADDI, AIDAI and scores of quality of life and functional ability in patients over time is needed to address the above-mentioned issues.

Besides the strong correlation between the ADDI and PGA-damage, the ADDI also moderately correlated to the PGA of disease activity. For a perfect discriminant validity, there would be no correlation between the ADDI and an activity score. However, in this case some degree of correlation is acceptable and probably unavoidable, since patients with more disease activity over the years generally accrue more damage. Furthermore, some items such as hearing loss may (initially) reflect both activity and damage. This overlap is partly prevented by the criterion that an item should be present for at least 6 months to be scored as a damage item. Therefore, disease activity has limited influence on the ADDI score.

Although the overall ICC was >0.8, the ICC of some individual items was less than 0.6. This could be explained by limited information provided in some of the paper cases, less experience of adult rheumatologists with paediatric measures (eg, scoring of pubertal delay) or the more subjective nature of some items (eg, musculoskeletal pain). Indeed, objective items such as hearing loss, renal insufficiency and osteoporosis all had an individual ICC of >0.8. As the overall ICC was good and the nature of the cases may be an important reason for a lower ICC, items scoring less than 0.6 were deemed acceptable, although sometimes with small alterations in the definition. A study testing the ADDI in real-life patients and also by individuals not involved in its development would be needed to overcome the above-mentioned issues.

During the face-to-face meeting, it was suggested to omit musculoskeletal pain from the ADDI, as it seems to be more subjective than the other items. Musculoskeletal pain, and other less objectively scored items such as fatigue and headache, might better be captured by patient-reported outcome measurements (PROM) in addition to the ADDI. A combination with (items from) the juvenile autoinflammatory disease multidimensional assessment report (JAIMAR) is worth considering.^26^ However, the JAIMAR is only validated on patients with FMF. Because musculoskeletal pain was emphasised by the patient representatives during the development phase of the ADDI as an important long-term disease burden in their daily activities, it was decided to keep this item in the ADDI, at least until a composite damage assessment including internationally validated PROMs is available.

As we found a relatively high ICC for the PGA-damage among the experts, one could argue that a detailed damage index is not necessary when the PGA is also reliable. However, we would still recommend the use of a damage index, since the physicians scoring the ADDI were considered experts in the area of AIDs, therefore their estimation of damage might be more accurate than that of physicians with less experience. Second, even though the estimates of PGA-damage might be reliable, an estimate of damage on a numerical scale does not give transparent information on why a certain amount of damage was estimated for a patient. The ADDI provides insight to the reasons why a certain level of damage is scored for a patient. Third, the ADDI provides a useful aide memoire and systematic means of collecting and quantifying damage, which is crucial to enable future comparisons between different studies.

Since damage prevention is one of the main purposes in the anti-inflammatory treatment of AIDs, its reliable assessment is an important measure in clinical practice as well as in therapeutic trials. As more information becomes available for the long-term outcomes of AIDs, the ADDI will have to reflect these in a data-driven manner. So far, it can be considered a reliable tool to assess disease damage for the four most commonly encountered monogenic AIDs.

## Supplementary Material

Ter Haar et al supplement

## Figures and Tables

**Table 1:** Intraclass correlation coefficient (ICC) of the total ADDI score in all patients (overall), the ICC of the ADDI in the four different diseases and separate ICCs for each category and damage item. Numbers in brackets indicate the 95% CI

	ICC (95% CI)
**Overall**	0.84 (0.78 to 0.89)
**Per disease**	
CAPS	0.82 (0.71 to 0.91)
TRAPS	0.62 (0.39 to 0.80)
FMF	0.84 (0.72 to 0.92)
MKD	0.73 (0.55 to 0.86)
**Per category**	
**Reproductive**	0.67 (0.59 to 0.76)
Sub/infertility	0.72 (0.63 to 0.79)
Amenorrhea	0.57 (0.46 to 0.67)
**Renal/amyloidosis**	0.88 (0.84 to 0.92)
Amyloidosis	0.76 (0.69 to 0.82)
Proteinuria	0.80 (0.73 to 0.85)
Renal insufficiency	0.84 (0.78 to 0.88)
**Developmental**	0.54 (0.43 to 0.64)
Growth failure	0.57 (0.46 to 0.67)
Puberty delay	0.29 (0.16 to 0.43)
**Serosal**	0.64 (0.54 to 0.72)
Serosal scarring	0.64 (0.54 to 0.72)
**Neurological**	0.75 (0.67 to 0.81)
Developmental delay	0.48 (0.37 to 0.60)
Cognitive impairment	0.54 (0.43 to 0.65)
Elevated ICP	0.65 (0.56 to 0.74)
CNS involvement	0.67 (0.58 to 0.75)
**Ears**	0.86 (0.82 to 0.90)
Hearing loss	0.86 (0.82 to 0.90)
**Ocular**	0.74 (0.66 to 0.80)
Ocular damage	0.74 (0.66 to 0.80)
**Musculoskeletal**	0.73 (0.64 to 0.80)
Joint restriction	0.52 (0.41 to 0.63)
Bone deformity	0.74 (0.66 to 0.81)
Osteoporosis	0.86 (0.81 to 0.90)
Musculoskeletal pain	0.47 (0.35 to 0.58)

ADDI, Autoinflammatory Disease Damage Index; CAPS, cryopyrin-associated periodic syndrome; CNS, central nervous system; FMF, familial Mediterranean fever; ICP, intracranial pressure; MKD, mevalonate kinase deficiency; TRAPS, tumour necrosis factor receptor-associated periodic syndrome.

**Table 2:** Definitive ADDI including glossary of terms

Damage item	Grading*	Points†
Reproductive		*Max 2*
Sub/infertility		2
Amenorrhea		1
Renal/amyloidosis		*Max 6*
Amyloidosis	Limited/extensive^1^	2/3
Proteinuria		1
Renal insufficiency	Moderate/severe^2^	2/3
Developmental		*Max 3*
Growth failure		2
Puberty delay		1
Serosal		*Max 1*
Serosal scarring		1
Neurological		*Max 6*
Developmental delay		2
Cognitive impairment		3
Elevated intracranial pressure		2
Central nervous system involvement		3
Ears		*Max 2*
Hearing loss	Moderate/severe^3^	1/2
Ocular		*Max 3*
Ocular involvement	Mild/moderate/severe^4^	1/2/3
Musculoskeletal		*Max 4*
Joint restriction		2
Bone deformity		2
Osteoporosis		1
Musculoskeletal pain		1

The total ADDI score is the sum of the eight categories (maximum 27 points).

* Grading: scoring depends on the severity of damage:

1 Amyloidosis: limited, affecting one organ extensive, affecting more than one organ.

2 Renal insufficiency: moderate, glomerular filtration rate (GFR) between 15 and 60 mL/min/1.73 m^2^; severe, GFR <15 mL/min/1.73 m^2^, dialysis or transplantation.

3 Hearing loss: moderate, hearing impairment without requirement of hearing aids or a cochlear implant; severe, hearing impairment requiring hearing aids or a cochlear implant.

4 Ocular involvement: mild, ocular damage without visual impairment; moderate, with visual impairment; severe, legal blindness.

† Points are given when the item is present. For items with grading in severity, the lowest score is given for mild involvement and the highest for severe involvement. For each category, the score is limited to a specific maximum.

ADDI, Autoinflammatory Disease Damage Index; Max, maximum.

Glossary of terms

*Amenorrhea:* Primary amenorrhea: absence of menarche at the age of 16 years or absence of menarche 5 years after the larche in a woman. Secondary amenorrhea: absence of the menses for six consecutive months or more in a woman who previously had menstrual cycles.

*Amyloidosis:* Symptomatic amyloidosis confirmed by examination of tissue sections by Congo red dye or serum amyloid P component (SAP) scintigraphy.

*Bone deformity:* Bone deformation or overgrowth on clinical examination and/or imaging studies.

*Central nervous system involvement:* Focal deficits (gross and/or fine sensorimotor), diffuse deficits (eg, memory, behaviour), seizures and spinal cord symptoms. Neuropsychiatric disorders unrelated to the disease should not be scored.

*Cognitive impairment:* Requirement of special education because of cognitive impairment or IQ below 70 as defined by neuropsychological assessment (eg, Wechsler Intelligence Scale for Children, WISC) or other age-appropriate equivalents.

*Developmental delay*$: Failure to reach age-appropriate developmental milestones, including language/speech, motor, social/emotional, and cognitive milestones.

*Elevated intracranial pressure:* Signs and/or symptoms of elevated intracranial pressure supported by appropriate techniques#.

*Growth failure:* Defined as the presence of at least two of the three features:
Lower than the third percentile or −2 SD height for age. Growth velocity over 6 months lower than the third percentile or −2 SD for age. Crossing at least two centiles (5%, 10%, 25%, 50%, 75%, 90%, 95%) on growth chart.

For patients older than 18 years: Pathological short stature (eg, below third percentile or −2 SD for normal ethnic population).

*Hearing loss:* Sensorineural hearing impairment of better ear, confirmed by audiometry or another age-appropriate technique, or requirement of hearing aids or a cochlear implant.

*Infertility:* A disease of the reproductive system defined by the failure to achieve a clinical pregnancy after 12 months or more of regular unprotected sexual intercourse, not due to known disorders in the unaffected partner.

*Joint restriction:* Fixed limitation in the normal range of motion of joints affecting function, with or without destructive arthropathy or avascular necrosis.

*Musculoskeletal pain:* Non-inflammatory musculoskeletal pain impairing activities of daily living.

*Ocular involvement:* Ocular damage (eg, optic nerve atrophy, elevated intraocular pressure or cataract) of better eye, documented by an ophthalmologist, with or without visual impairment.

*Osteoporosis:* Reduced bone mineral density with vertebral collapse and/or pathological fractures confirmed with imaging, which may include bone densitometry. Requires both evidence of decreased bone density and fracture, ‘low bone density’ by itself is insufficient.

*Proteinuria:* Persistent urinary protein-to-creatinine ratio of >20 mg/mmol in the first morning void; and/or a daily protein excretion of >0.3 g/24 hours, or urine albumin-to-creatinine ratio of >15 mg/mmol.

*Puberty delay:* A Tanner stage below −2 SDs for age or below the third percentile for age or any Tanner stage after pharmacological induction of puberty.

*Renal insufficiency:* Glomerular filtration rate (GFR) of <60 mL/min/1.73 m^2^, dialysis or transplantation.

*Serosal scarring:* Symptomatic adhesions or fibrosis affecting pericardium, pleura, peritoneum and/or retroperitoneum, supported by imaging techniques, endoscopy or surgery.

$Only for paediatric patients; #such as funduscopy, neuroimaging or lumbar cerebrospinal fluid (CSF) pressure measurement.
