# Supplementary material for: In silico validation of the Autoinflammatory Disease Damage Index
Source: Ann Rheum Dis. Author manuscript; Available in PMC 2021 Sep 2. (PMC8411437; doi:10.1136/annrheumdis-2018-213725)
Supplement: Ter Haar et al supplement [file NIHMS1736033-supplement-Ter_Haar_et_al_supplement.docx]

**SUPPLEMENTARY TABLES**

Index

1. Cronbach’s alpha – inter-item correlation matrixes
2. Reproductive
3. Renal/amyloidosis
4. Developmental
5. Neurological
6. Musculoskeletal
7. Overview of comments explaining discrepancies and comments discussed in final survey and face-to-face meeting.

**1. Cronbach’s alpha**

*Not applicable if a category consists of less than 2 items*

**a. Reproductive** (Cronbach’s alpha 0.27)

|  | **Amenorrhea** | **Infertility** | **Cronbach’s alpha if item deleted** |
| --- | --- | --- | --- |
| **Amenorrhea** | - | 0.23 | *not applicable* |
| **Infertility** | - | - | *not applicable* |

**b. Renal/amyloidosis** (Cronbach’s alpha 0.78)

|  | **Amyloidosis** | **Proteinuria** | **Renal insufficiency** | **Cronbach’s alpha if item deleted** |
| --- | --- | --- | --- | --- |
| **Amyloidosis** | - | 0.59 | 0.60 | 0.71 |
| **Proteinuria** | - | - | 0.73 | 0.75 |
| **Renal insufficiency** | - | - | - | 0.60 |

**c. Developmental** (Cronbach’s alpha 0.23)

|  | **Growth failure** | **Puberty delay** | **Cronbach’s alpha if item deleted** |
| --- | --- | --- | --- |
| **Growth failure** | - | 0.25 | *not applicable* |
| **Puberty delay** | - | - | *not applicable* |

**d. Neurological** (Cronbach’s alpha 0.75)

|  | **Developmental delay** | **Elevated intracranial pressure** | **Cognitive impairment** | **Central nervous system involvement** | **Cronbach’s alpha if item deleted** |
| --- | --- | --- | --- | --- | --- |
| **Developmental delay** | - | 0.34 | 0.66 | 0.35 | 0.70 |
| **Elevated intracranial pressure** | - | - | 0.43 | 0.48 | 0.73 |
| **Cognitive impairment** | - | - | - | 0.49 | 0.63 |
| **Central nervous system involvement** | - | - | - | - | 0.72 |

**e. Musculoskeletal** (Cronbach’s alpha 0.51)

|  | **Musculoskeletal** | **Joint restriction** | **Osteoporosis** | **Bone deformity** | **Cronbach’s alpha if item deleted** |
| --- | --- | --- | --- | --- | --- |
| **Musculoskeletal** | - | 0.25 | -0.002 | 0.15 | 0.50 |
| **Joint restriction** | - | - | 0.14 | 0.39 | 0.29 |
| **Osteoporosis** | - | - | - | 0.22 | 0.53 |
| **Bone deformity** | - | - | - | - | 0.32 |

**2. Overview of comments explaining discrepancies and comments discussed in final survey and face-to-face meeting.**

| **Comment** | **N** | **Changes in definition or scoring** |
| --- | --- | --- |
| Unclear case description/insufficient information / different interpretation of information between observers | 73 | Na |
| Difficulties scoring puberty delay due to pharmacological induction of puberty | 3 | Addition of ‘…or below the 3^rd^ percentile for age or any Tanner stage after pharmacological induction of puberty’ to definition |
| Difficulties scoring serosal scarring | 7 | Addition of ‘Symptomatic…’ to definition |
| Difficulties differentiating developmental delay/cognitive impairment from CNS and or on how to interpret psychiatric disorders | 6 | Addition of ‘ Neuropsychiatric disorders unrelated to the disease should not be scored’ to definition |
| Unclarities considering definitions of joint restriction | 2 | Addition of ‘…affecting function…’ to definition |
| Suggestion: more points for end stage renal disease | 1 | No |
| Suggestion for higher score for growth failure | 1 | No changes in scoring, but addition of ‘-2SD’ to definition |
| Maximum score of the ADDI is always lower in males than in females because of the item amenorrhea | 1 | Total scoring of reproductive changed to 2 |
